# Mechanisms of disturbed emotion processing and social interaction in borderline personality disorder: state of knowledge and research agenda of the German Clinical Research Unit

**DOI:** 10.1186/2051-6673-1-12

**Published:** 2014-09-09

**Authors:** Christian Schmahl, Sabine C Herpertz, Katja Bertsch, Gabriele Ende, Herta Flor, Peter Kirsch, Stefanie Lis, Andreas Meyer-Lindenberg, Marcella Rietschel, Miriam Schneider, Rainer Spanagel, Rolf-Detlef Treede, Martin Bohus

**Affiliations:** Department of Psychosomatic Medicine and Psychotherapy, Central Institute of Mental Health Mannheim, Medical Faculty Mannheim / Heidelberg University, J 5, 68159 Mannheim, Germany; Department of General Psychiatry, Center of Psychosocial Medicine, Medical Faculty Heidelberg / Heidelberg University, Heidelberg, Germany; Department of Neuroimaging, Central Institute of Mental Health Mannheim, Medical Faculty Mannheim / Heidelberg University, Mannheim, Germany; Institute of Neuropsychology and Clinical Psychology, Central Institute of Mental Health Mannheim, Medical Faculty Mannheim / Heidelberg University, Mannheim, Germany; Department of Clinical Psychology, Central Institute of Mental Health Mannheim, Medical Faculty Mannheim / Heidelberg University, Mannheim, Germany; Department of Psychiatry and Psychotherapy, Central Institute of Mental Health Mannheim, Medical Faculty Mannheim / Heidelberg University, Mannheim, Germany; Department of Genetic Epidemiology in Psychiatry, Central Institute of Mental Health, Medical Faculty Mannheim, Heidelberg University, Mannheim, Germany; Institute of Psychopharmacology, Central Institute of Mental Health, Medical Faculty Mannheim / Heidelberg University, Mannheim, Germany; Department of Neurophysiology, Center for Biomedicine and Medical Technology Mannheim, Medical Faculty Mannheim / Heidelberg University, Mannheim, Germany

**Keywords:** Borderline personality disorder, Emotion processing, Social interaction, Neuroimaging, Genetics

## Abstract

The last two decades have seen a strong rise in empirical research in the mechanisms of emotion dysregulation in borderline personality disorder. Major findings comprise structural as well as functional alterations of brain regions involved in emotion processing, such as amygdala, insula, and prefrontal regions. In addition, more specific mechanisms of disturbed emotion regulation, e.g. related to pain and dissociation, have been identified. Most recently, social interaction problems and their underlying neurobiological mechanisms, e.g. disturbed trust or hypersensitivity to social rejection, have become a major focus of BPD research. This article covers the current state of knowledge and related relevant research goals. The first part presents a review of the literature. The second part delineates important open questions to be addressed in future studies. The third part describes the research agenda for a large German center grant focusing on mechanisms of emotion dysregulation in BPD.

## Current state of knowledge

Borderline Personality Disorder (BPD) typically begins during adolescence, shows a peak of symptom severity during young adulthood, and appears to decline modestly but steadily over the years. A large-scale prospective study conducted in the US [1], which is currently viewed as the most valid, showed remission rates of 86% after a 10-year follow-up (defined as a minimum of 4 years not meeting DSM-IV BPD criteria). These findings have usually been interpreted as indicating that most borderline patients attain a reasonably good overall outcome 10 years after the index admission. However, a more recent analysis of long-term social integration of this population showed that only 50% of patients achieved both remission from BPD symptoms and good social and vocational functioning for a minimum of 2 years [2]. Good social functioning was defined as having a score of at least 61 on the Global Assessment of Functioning (GAF) scale. Of those who achieved recovery, 34% later relapsed, resulting in a 10 years total recovery rate of about 35%.

These long-term data are based on samples assessed in the US starting in the 1990s, a time when development of disorder-specific treatments was in its infancy. It might be argued that the relatively low recovery rates are due to a lack in specific psychiatric care management. Within the last decade, a number of specific psychosocial treatments with evidence of both effectiveness and efficacy have been developed (for overview, see [3]), and most of these treatments show medium to large effect sizes in the reduction of typical borderline behavior or psychopathology. However, social integration of BPD patients continues to be problematic: up to 50% of treated patients have a GAF score lower than 60, indicating persistent serious social problems that may correspond to the long-term follow-up data cited above. One possible interpretation of these data is that social integration of patients with BPD (and thus recovery) is impaired by factors distinct from those targeted by the currently existing best practice treatments. If so, this necessitates further research into the underlying pathophysiology, which we propose below may be linked to emotional dysregulation, and the transfer of those findings into a new generation of psychotherapeutic interventions. We will describe central mechanisms of BPD in the following; it should be noted that these do not necessarily correspond to the Research Domain Criteria suggested by the NIH.

### Phenomenology of emotion processing in BPD

A confirmatory factor analytic study of BPD [4] revealed three core domains of psychopathology: affective dysregulation, interpersonal disturbances, and behavioral dysregulation. Three recent large twin studies have all pointed to a common pathway model with one highly heritable general BPD factor [5–7]. The last two of these revealed two additional common liability factors, mainly reflecting affective and interpersonal dimensions. Currently, there is an ongoing debate about the hierarchy of these domains and their potential interactions [8]. From a sociobiological point of view, most of the interpersonal problems of BPD patients (such as rejection sensitivity, difficulties in cooperation, and hostile behavior) can be seen as being driven by dysfunctional emotion processing. From a developmental point of view, aversive interpersonal experiences (such as insecure primary care, early loss, or sexual abuse) can be seen as leading to dysfunctional social cognitions that induce problems in emotion regulation. However, rather than discussing these interactions as a chicken-and-egg problem, one can see them as circular mechanisms. According to an established model by Gross [9], emotion regulation in general includes not only social assumptions and neural regulatory mechanisms but also implicit action tendencies and social interaction.

Experienced clinicians have long suggested affective dysregulation as a core feature of BPD and a driving force behind several serious dysfunctional behavioral patterns [10–14]. Affective dysregulation is related to the frequency with which patients use maladaptive strategies to regulate affect [15] and predicts other BPD behaviors [14], including suicidal ideation and suicide attempts [16, 17], maladaptive interpersonal behaviors, and impulsive coping behaviors [18] such as alcohol abuse [19].

Several recent empirical studies have confirmed these clinically based observations. It has been demonstrated that, under both daily life and experimental conditions, BPD patients experience significantly more intense aversive emotions, higher tension, and more volatility of mood than do healthy controls [20–23]. In particular, ambulatory assessment (AA), the favored assessment methodology for acquiring data under natural conditions, has provided insight into the phenomenology of affective instability that is present in BPD. Several studies based on AA have yielded consistent findings, revealing an overall heightened affective instability of patients with BPD compared with healthy controls (for an overview, see [24]). It should be stated that these patterns of affective instability do not seem to be specific for BPD. As Santangelo and coworkers [25] recently showed, similar patterns have been found in samples of patients with posttraumatic stress disorder (PTSD) or with binge eating disorder.

When it comes to distinct and specific emotions in BPD, social emotions such as shame, guilt, disgust, and the fear of social rejection seem to be the most important [26–30] (Domsalla M, Liebke L, Thome J, Haeussler K, Bohus M, Lis S: Rejection Sensitivity and Symptom Severity in Patients with Borderline Personality Disorder: Effects of Childhood Maltreatment and Self-Esteem. In Borderline Personality Disorder and Emotion Dysregulation.; 2014. submitted). According to both clinical and scientific perspectives, the experience of high levels of aversive tension often engenders marked dissociative symptoms, which in turn are related to hypoalgesia [31, 32]. Emotional learning may be largely inhibited during dissociative states [33]. Dysfunctional emotion regulation may lead to strong aversive tension in line with dissociative experience, which in turn hinders adequate emotional learning. There is strong evidence that non-suicidal self-injury (NSSI), a phenomenon very often observed in BPD, is mostly used to down-regulate these states of aversive tension or to stop dissociative states [34–36].

Before we discuss the distinct mechanisms of dysfunctional emotion processing in BPD, a short overview is given on the current taxonomy of emotion processing in general.

### Basics of emotion processing

From a socio-psychological perspective, emotions may be thought of as complex and evolved patterns of response to both external and internal stimuli, providing a fast situational interpretation along with a corresponding action tendency. Emotion processing involves automatic and intentional processes that influence the occurrence, intensity, duration, and expression of emotions. There is a wide spectrum of theories on how emotions can influence self-theory, identity, decision-making, social interaction, and even policy (for overview, see [37]).

Among others, Gross and colleagues [9, 38] have proposed a model of emotion regulation, based on influential emotion theorists (e.g., [39–41]), which emphasizes the explicit or implicit appraisal of external or internal emotional cues that trigger a set of experiential, physiological, and behavioral response tendencies. This model has already influenced treatment development for BPD [42] and might serve as a current basis for research. According to this model, emotions can be modulated automatically or by either manipulating the input to the system (*antecedent-focused emotion regulation strategies*) or by manipulating the output of the regulation process (*response-focused emotion regulation strategies*). Antecedent-focused strategies include both implicit and explicit strategies, such as situation selection or modification and cognitive techniques (e.g., reappraisal, attention deployment, or reframing of the situation), while response-focused strategies include both implicit and explicit strategies that can be subdivided into physiological, cognitive, and behavioral processes.

However, Gross’s model does not consider the potential role of emotional awareness or experiential avoidance in emotion regulation. One could argue that emotion regulation is mostly an automatic process, independently of cognitive meta-awareness. On the other hand, recent research has clearly demonstrated the potential role of experiential avoidance in the pathogenesis of psychic disorders. Experiential avoidance (EA) not only includes any behavior that seeks to avoid, or escape from, unwanted internal experiences or those external conditions that elicit them, but also the pure awareness of activated emotions [43]. Consequently, increasing emotional awareness and emotion acceptance is currently seen as an important mode of action in psychotherapy in general and in BPD [44]. Schramm and coworkers [45] showed that borderline personality features were associated with significantly higher levels of EA and difficulties in emotion regulation. Hierarchical regression analyses showed that EA made a small but significant incremental and independent contribution to borderline features when added to a model that already included difficulties in emotion regulation.

On a neuroanatomical level, the central areas involved in the “emotion regulation circuitry” are thought to be the dorsolateral and ventral areas of the prefrontal cortex (including the anterior cingulate cortex [ACC]), as well as the amygdala, the hippocampus, and the insula [38]. It should, however, be stressed that these regions fulfil several functions besides emotion regulation as well. Ochsner and Gross suggested a psychobiological circular model of emotion processing whereby emotions are generated and modulated by interplaying macro- and micro-circuits of “bottom-up” and “top-down” processes. According to this model, central areas such as the amygdala and the insula are involved in the evaluation of external and internal stimuli regarding their emotional valence. These stimuli are further processed in the hypothalamus and in brain stem regions in order to activate autonomic and behavioral responses. In parallel, prefrontal and parietal cortical areas serve to allocate attention and to activate potential behavioral responses. Regulatory processes associated with areas of the lateral and medial prefrontal cortex (MPFC) act to control and modulate emotional activation, thereby covering typical response-focused regulation strategies. Recent studies suggest a regulatory hierarchy, whereby the dorsolateral prefrontal cortex (DLPFC) and areas of the anterior medio-prefrontal cortex modulate the cingulate, which in turn modulates the amygdala and further subcortical areas [46, 47].

Importantly, these regulatory interactions are sensitive to genetic variation in candidate genes that have been reported to have an impact on personality as well as on the risk for affective disorders and for which gene-environment interactions with early childhood trauma have been found [48–50]. Recent work has extended these results to genome-wide significant risk variants for severe psychiatric disorder [51] and has suggested a common circuitry for emotion regulation and extinction on which genetic and environmental risk factors converge [52]. Regulatory processes can also be activated by cognitive reappraisal, by changing attention, or by activating memories [50]. These cognitive strategies result in an activation of lateral and medial prefrontal areas which, in turn, involve the ACC and ultimately dampen emotional arousal by attenuating the activity of the amygdala, the mid-cingulate, and areas of the insula (for review, see [50]). It should be stressed here that mechanisms of emotion regulation are subject to genetic variation, to maturing processes, and to inter-individual variation, as well as to environmental risk factors such as early adversity or poverty. Notably, during adolescence, there appears to be a marked imbalance between increased sensitivity and susceptibility of subcortical limbic areas to emotional stimuli and not yet fully mature prefrontal areas. This imbalance may account for the tendency towards high emotional activation and impulsivity during adolescence in general (reviewed in [53–55]).

### Pathophysiology of emotion processing in BPD

This section will give an overview about findings in the fields of genetics, structural imaging and spectroscopy, functional imaging, pain processing and dissociation, as well as neurochemistry.

#### Genetics of BPD

To date, the only studies on the genetics of BPD have been small-scale and have yielded mostly inconsistent results. A recent meta-analysis [56] found no direct role of more than 20 investigated genetic variants in BPD or BPD traits. No associations between BPD traits and three serotonergic polymorphisms, two common polymorphisms of the serotonin transporter gene (SCL6A4), the promotor insertion/deletion (5-HTTLPR) and the intron 2 VNTR, or the rs1800532 polymorphism of the tryptophan hydroxylase 1 gene (TPH1) were found. All studies, however, were conducted with very small sample sizes, and to date there has been no published genome-wide association study in BPD. On the other hand, a genome-wide analysis and replication study of borderline personality features [57] has been conducted in three Dutch cohorts, which were comprised of a total of 8426 participants. While no genome-wide significant association was identified, the top findings which could be replicated in the independent replication sample were located in a region corresponding to SERINC5*,* a protein involved in myelination.

More promising avenues of study might use gene x gene (e.g., [58, 59] and gene x environment approaches (e.g., [60] interactions). Interestingly, Distel and coworkers [61] were able to demonstrate that the impact of genetic factors on BPD features is lower in individuals who have been victims of sexual abuse than in those who have experienced other serious life events. Epigenetic research in BPD is also just beginning [62]. A pilot study on mRNA gene expression demonstrated no effect of BPD per se, but an effect of dissociation on the expression of genes involved in immune system regulation as well as cellular signalling/second-messenger systems [63].

#### Brain structure

(*In this paragraph, the following annotations for replication were included: A = replicated by more than one research group, B = replicated by members within the same research group, C = single study only without replication).

Among the most robust findings of structural imaging in BPD patients are reduced volumes of the amygdala, the hippocampus, the OFC, and the ACC [64, 65] (A*). The most recent meta-analysis, which incorporated 11 studies with 205 BPD patients and 222 healthy controls, showed an average decrease of 11% in the size of the hippocampus and of 13% in the size of the amygdala [66]. Another meta-analysis demonstrated that hippocampal reductions are more pronounced in BPD patients who have co-morbid PTSD. In those without PTSD, right but not left hippocampal volumes were reduced [67]. More recently, a whole-brain voxel-based morphometry (VBM) study in 60 patients with BPD confirmed volume reduction in the hippocampus and amygdala as well as in the fusiform and cingulate gyri [68]. A further study [69], which enrolled 30 BPD patients and 33 controls, found the gray matter of patients with BPD to be reduced in the hippocampus but increased in the hypothalamus compared to healthy participants. Hypothalamic volume correlated positively with the history of traumatization in BPD patients [70] (C*). Another VBM study demonstrated that BPD and bipolar disorder have relatively distinct patterns of structural alterations, with mostly fronto-limbic alterations in BPD [71] (C*), however these data need replication within a larger sample. Reduced volumes of the left ACC and the right OFC (but not of the amygdala or hippocampus) were detectable in a first study including adolescent BPD patients (average age 17.4 years), and were found to correlate with impulsivity and non-suicidal self-injury [72, 73] (C*). Specific differences were also found between criminal offenders with either BPD or psychopathy [74] (C*). The BPD offenders had lower volumes in the orbitofrontal and ventromedial prefrontal cortex regions subserving emotion regulation and reactive aggression, while, for psychopathic offenders, the most significant volumetric reductions were in the midline cortical areas involved in the processing of self-referential information and self-reflection.

Ruesch and coworkers [75] (C*) used diffusion tensor imaging (DTI) to investigate the relationship between white matter integrity in the inferior frontal cortex and several core symptoms of BPD, as well as to measure the neuropsychological performance of BPD patients with co-occurring attention-deficit-hyperactivity disorder (ADHD). Initial evidence was found for a possible relationship between core symptoms of BPD and structural alterations of the white matter in the inferior frontal cortex, as the average diffusion in the inferior frontal cortex was related to affective dysregulation, anger/hostility, and dissociation. However, no significant group differences were detected in the DTI measurements between BPD patients and healthy controls. Additional analyses of this sample revealed abnormalities of inter-hemispheric connectivity between both sides of the anterior cingulate [76] (C*). Further DTI studies showed decreased fractional anisotropy (FA) in the genu and rostral areas of the corpus callosum as well as in left and right prefrontal white matter [77] (C*) in BPD adults and in the fornix in BPD adolescents [78] (C*). New and coworkers [79] (C*) found decreased FA in the inferior longitudinal fasciculus and other areas in BPD adolescents but not in BPD adults. This most probably speaks for a transient disturbance of connectivity in the development of BPD.

According to two MR spectroscopy study, the absolute concentration of N-acetyl aspartate (NAA) in the DLPFC is reduced by 19% in BPD patients, suggesting a reduced cell density or a functional impairment within this region [80] (C*). Compared with healthy controls, subjects with BPD were found to have reduced NAA concentrations in the amygdala [81] (C*) and significantly higher levels of glutamate in the ACC [82] (B*). In the latter study, which could recently be confirmed in a new sample (Ende, Cackowski, van Eijk, Sack, Sobanski, Krause-Utz, Schmahl: Impulsivity and aggression are differentially associated with anterior cingulate glutamat and GABA concentrations in Borderline Personality Disorder and Attention-Deficit-Hyperactivity Disorders. In preparation), a positive correlation between glutamate concentration and self-rated impulsivity was observed, as well as between glutamate concentration and dissociation scores.

#### Neurochemistry

Initial studies on the neurochemistry of BPD primarily focused on the hypothalamic–pituitary–adrenal (HPA) axis as well as on the serotonergic, glutamate, oxytocin, and opioid systems.

#### HPA axis

Findings with respect to the HPA axis are inconsistent, possibly due to confounding effects of co-occurring disorders such as depression and PTSD and small sample sizes. While one study reported increased salivary cortisol levels under daily life conditions [83], another found a significantly reduced cortisol response to experimentally induced social stress [84]. Notably, in this study, the reduced cortisol response was not paralleled by reduced ACTH secretion, which might suggest a stress-associated hypo-activity of the adrenal cortex. A similar hypo-activity in response to the Trier Social Stress Test (TSST) was found in adolescents with NSSI, nearly half of whom fulfilled diagnostic criteria for BPD [85]. In contrast to healthy controls, cortisol administration enhanced rather than impaired memory retrieval in BPD patients [86], which is similar to the effects it has on PTSD patients [87].

#### Serotonin

This neurotransmitter system has important regulatory functions in frontro-striatal circuits, and dysfunctions which are considered to be important predictors for impulsive behavior [88, 89]. Subjects with impulsive and aggressive behavioral tendencies were consistently found to have reduced cerebrospinal fluid (CSF) concentration of the metabolite 5-HIAA and reduced neuroendocrinological reaction to serotonergic agonists (e.g., D- or D,L-fenfluramine, meta-chlorophenylpiperazine) [90, 91]. Neuroimaging studies, each conducted in only 8 BPD patients (e.g., [92, 93]), have revealed altered metabolism at baseline as well as in response to serotonergic challenge in prefrontal regions including the ACC. SSRI treatment (fluoxetine 20 mg/day) normalized prefrontal cortex dysfunction in impulsive-aggressive BPD patients in one study. [94].

#### Oxytocin

As a key mediator of trust behavior, the neuropeptide oxytocin (OT) is involved in attachment and pro-social behavior [95]. Furthermore, it has been shown that OT modulates stress responses, especially in social contexts, and may be affected by early life adversity [96]. Parents reporting greater parental care showed higher plasma OT, low-risk CD38 alleles, and more physical contact with their infants [97]. On a neurobiological level, there is rising evidence that the application of OT might attenuate the response of the amygdala to emotional stimuli [98]. The specific circuit on which OT has an impact is a convergence zone for genetic and environmental risk [52]. Thus from a developmental as well as a neurobiological point of view, it appears plausible that alterations of the OT system might be involved in the pathophysiology of BPD [99]. Correspondingly, Bertsch and coworkers [100] reported decreased peripheral OT concentrations in female BPD patients, which negatively correlated with severity of early traumata. Interestingly, a pilot study [101] found that intra-nasal application of OT *impaired* trustful expectations in BPD subjects, showing that while the OT system seems to be disturbed in BPD, a simple substitution of the neuropeptide may not fix the problem. Bertsch and coworkers [102] found a normalization of abnormal behavioral and neuronal patterns after intranasal OT application while BPD patients were scanning angry faces, suggesting that OT may decrease threat hypersensitivity in this group of patients.

#### Opioids

Attenuated pain perception and the lack of effective emotion regulation in BPD implicate a potential dysfunction of the endogenous opioid system [99, 103]. BPD patients with NSSI behavior were found to have significantly lower levels of CSF β-endorphin and met-enkephalin when compared with a non-NSSI BPD group [104]. A PET study [105] found that under neutral conditions, BPD patients compared to controls showed greater regional μ-opioid receptor availability bilaterally in the orbitofrontal cortex, caudate, nucleus accumbens, and left amygdala but lower μ-opioid receptor availability in the posterior thalamus; whereas during emotion induction (sadness), they showed greater activation of the endogenous opioid system in the pregenual anterior cingulate, left orbitofrontal cortex, left ventral pallidum, left amygdala, and left inferior temporal cortex.

#### Brain function and networks

(*In this paragraph, the following annotations for replication were included: A = replicated by more than one research group, B = replicated by members within the same research group, C = single study only without replication).

The cerebral processing of emotional stimuli in BPD patients has been investigated in several PET and fMRI studies. One study [106] found bilateral amygdala hyperactivity in BPD patients while viewing emotionally aversive pictures. This finding has been repeatedly replicated with elevated amygdala responses to neutral pictures as well [107–110] (A*). A recent finding of decreased amygdala habituation in response to repeatedly presented stimuli is consistent with the clinical observation of abnormally strong and long-lasting reactions to emotional cues [111] (C*). Functional imaging studies using cues or scripts to induce BPD-specific characteristics are indicative of disturbed functioning in prefrontal regions. For instance, script-driven induction of traumatic events or of social separation in traumatized female BPD patients and traumatized women not meeting BPD criteria resulted in a lower activation of both the ACC and the OFC in the former [112, 113] (B*). Studies on functional correlates of response inhibition have yielded further evidence for functional impairments of prefrontal areas, notably of the DLPFC, the rostral ACC, and the OFC [114] (C*). Minzenberg and coworkers [115], using an implicit affect regulation task (responses to threatening vs. neutral faces), demonstrated specifically enhanced neural activation of the right amygdala in BPD along with attenuated activations of the rostral ACC. In response to an explicit affect regulation task based on reappraisal strategies, OFC hypoactivation, in addition to insular hyperactivation, was found, while negative emotions were down-regulated [109] (C*). In a study that used attentional distraction as a further regulation task, BPD patients were shown to exhibit higher left-sided amygdala activation than healthy controls [116] (C*). Reduced connectivity between the OFC and the amygdala in BPD patients was reported by New and coworkers [117] (C*). Interestingly, in a pilot study, successful psychotherapy was found to be paralleled by normalization in the amygdala response as well as in prefrontal top-down areas [118] (B*). These findings were recently corroborated in a larger controlled trial (Schmitt, Niedtfeld, Winter, Herpertz, Schmahl unpublished observations). A recent meta-analysis of fMRI studies across different stimulation methods revealed greater activity in the insula and reduced activation in the subgenual ACC and DLPFC in BPD patients as compared to controls [119] (A*).

#### Pain processing and dissociation

As already mentioned, reduced sensitivity to pain (hypoalgesia) and a close relationship between stress and hypoalgesia have been consistently reported in BPD patients [120–124]. The sensory-discriminative component in pain processing does not appear to be disturbed, but abnormalities have been detected for affective pain processing. More specifically, a deactivation of the amygdala has been found during pain induction in BPD patients [122, 125]. A modulation of the affective pain component in BPD patients by the well-known COMT val158met polymorphism has been demonstrated [126], however this findings needs replication. Furthermore, higher BPD symptom severity and trait dissociation were associated with an attenuated signal decrease of the default mode network in response to painful stimulation, and patients with BPD exhibited less posterior cingulate cortex connectivity with the left DLPFC during painful stimulation [127]. Preliminary evidence from recent studies indicates specificity of reduced pain sensitivity, as no differences in proprioception and exteroceptive sensitivity were found [123, 128]. Also, no differences between BPD patients and controls were found regarding interoception in a task where participants had to observe and count their own heartbeats [129].

In a study that tested the aspect of emotion regulation by sensory stimulation, pain that was experimentally induced by thermal stimuli was found to result in the attenuation of amygdala hyperactivity induced by affective pictures [108]. Functional connectivity analyses revealed normal inhibitory connectivity between the left amygdala and MPFC and between the right anterior insula and DLPFC when negative pictures were combined with painfully hot stimulation but not when they were combined with non-painfully warm stimulation [130], suggesting that there may be a specificity of painful stimuli in the context of sensory emotion regulation in BPD. Using incision-induced pain, which takes into account tissue damage and thus provides a more valid model for non-suicidal self-injury, a stress-reducing effect of an incision in the forearm in terms of reduced subjective arousal and increased heart rate variability could be demonstrated [131]. These findings were recently replicated in an fMRI study in which an additional restitution of post-stress amygdala-mPFC coupling following incision was shown (Reitz, Kluetsch, Niedtfeld, Knorz, Lis, Paret, Kirsch, Meyer-Lindenberg, Treede, Baumgaertner, Bohus, Schmahl: Incision and stress regulation in borderline personality disorder. neurobiological mechanisms of self-injurious behavior. Submitted).

It has been suggested that dissociation constitutes an emotional over-modulation mode that responds to the experience of (traumatic) stress, as opposed to an emotional under-modulation mode with predominantly intrusive symptoms and that these two modes can be segregated on a neurofunctional level [132]. In particular, over-activity of medial prefrontal brain regions with concomitant limbic down-regulation is hypothesized to underlie dissociative psychopathology. Corroboration of these assumptions comes from several sources. In one study, patients with high levels of dissociation were found to have significantly lower startle responses compared to those with low levels of dissociation [133], while another study found that dissociation scores were negatively correlated with activity in the amygdala, insula, and ACC during emotional distraction that took place while the participants were performing a working memory task [134]. Dissociative phenomena have some similarity with body-related illusions such as out of body experiences and it might therefore be useful to expand the research on these phenomena from nociception and pain processing to the processing of body perception, which has been shown to be altered in BPD [135] .

### Interpersonal disturbances

Interpersonal disturbances have been central to characterizations of BPD since the earliest descriptions of this disorder [136]. In the last few years, clinically based observations have been confirmed by empirical data that support alterations of the social lives in BPD. Romantic partnerships are characterized by high instability in the form of frequent breakups and reconciliations accompanied by low marital satisfaction, high attachment insecurity, communication problems, a high level of physical and psychological violence, and a tendency to choose partners who also have mental problems [137–139]. Several studies observed altered maternal behavior linked with impaired social interaction behavior in the children of BPD patients [140–142]. However, one should be careful about generalizing these premature findings, which are based on small and selective samples. In everyday life, BPD patients often experience more unstable social relations than healthy controls as well as fewer social interactions and have sometimes been shown to use maladaptive resources for social support within their social network [143, 144].

#### Social interaction in BPD

Social interaction problems have been investigated in BPD using questionnaires and experimental approaches that aim to measure behavior directly during natural or standardized interaction situations [145]. Subjective assessments in questionnaires point to BPD patients showing highly variable and more extreme interaction behaviors, which have been described as more hostile, more quarrelsome, and less affiliative in nature [143, 146]. However, it must be noted that findings based on self-based or observer-based ratings primarily reflect the perception of a subject’s competence, which might differ from their actual behavior during a social encounter.

In the last several years, an increasing number of studies have applied experimental approaches that measure interaction behavior in standardized situations within distinct social domains. Specifically, they have investigated dyadic or triadic interactions in the context of social exclusion, provocation of aggressive behavior, and the ability to display trustful and cooperative behavior (for review, see [145]).

#### Social rejection during social encounters

Several studies have used a virtual ball-tossing game called “cyberball” [147] to induce the experience of social rejection, which, together with intense fear of loss and abandonment, may constitute a central factor of interpersonal relationships in BPD. In this game, the co-players’ behavior is manipulated to mimic inclusion, exclusion, or neutral control conditions, during which the players act according to predefined rules; i.e., without personal motivations. In recently published studies, BPD patients were found to feel more excluded during the inclusion and neutral conditions [148–151]. The experience of social exclusion evoked especially intense negative emotions such as contempt, resentment, and anger, which were focused on others [150]. These findings suggest that the awareness of social exclusion and the resulting emotional reactions in BPD patients differ from those of healthy individuals. It is worth emphasizing that this seems to be true not only during the experience of social rejection but also during the inclusion and neutral situations. A recent fMRI-study linked these alterations to a lack in the modulation of activation in brain regions such as the insula and the precuneus depending on the nature of the social encounter [151].

No studies have yet addressed how BPD patients cope with the experience of social rejection, such as whether they tend to punish excluders or engage in self-protective strategies, as healthy individuals have been shown to do [152, 153]. Such studies may provide insight into the mechanism of dysfunctional social interaction behavior and may constitute the basis for the development of specific therapeutic intervention strategies.

#### Social-cognitive information processing

Social interaction behavior is based on a multitude of social cognitive processes, including the ability to recognize emotions or intentions in social partners. An increasing number of studies have confirmed that these processes are affected in BPD, although the exact nature of these alterations is still under debate. Studies on the decoding of facial expressions suggest that BPD patients show a heightened sensitivity to negative emotional cues [154–157]. However, other studies have shown impairments in detecting and labeling emotions for both negative and positive valent emotional expressions [158–162]. These heterogeneous findings point to the need to investigate which factors, such as social and non-social stress, emotion regulation abilities, dissociative symptoms, and the necessity of coordinating these functions with others in order to guide social interactions, modulate emotion recognition in BPD [163]. Empirical findings point to a social cognitive bias [164]. For example, Staebler and coworkers found that experimentally induced social rejection led to a negative bias for perceived social participation [165], while Barnow and coworkers [146] found that BPD patients have an interpretational bias which leads them to assume that others are hostile and angry. Similarly, Domes and coworkers [166] found a bias for recognizing angry facial expressions in ambiguous faces, a finding that was recently replicated in a larger sample (Izurieta, Bertsch, Herpertz unpublished). These data fit with studies that investigated trust and cooperation in exchange games established in behavioral economy. On a behavioral level, King-Casas and coworkers [167] used a multi-round trust game to demonstrate that BPD patients showed less generous behavior than healthy controls, leading to a breakdown in cooperation over the course of the interaction. Similarly, Unoka and colleagues [168] found that BPD patients failed to develop trust in an interaction partner during a multi-round trust game when they did not receive feedback about their partner’s behavior. These experimental data fit with the concept of BPD as a disorder of “mentalization”, which gave rise to the development of “Mentalization Based Therapy” – a BPD-specific psychosocial treatment which has proven to be effective [169, 170].

The term “mentalization” is derived from theory of mind, and concerns a complex cognitive and affective understanding of self and others and enables individuals to navigate effectively in the social world. However, while the concept of mentalization is a useful heuristic approach, it has been criticized as being too broad and multifaceted to be operationalized as a marker for specific BPD pathology [171]. An increasing number of experimental studies aim to precisely measure different components of mentalizing and its behavioral and cerebral alterations in BPD [163, 172–176]. In the future, these findings may allow identification of the components of mentalizing that are altered in BPD and, as a consequence, lead to further refinement of theoretical concepts and corresponding therapeutic interventions.

### Animal models

Animal models of neuropsychiatric disorders are a valuable tool and an indispensable part of neurobiological and psychiatric research that might even have direct relevance as biomarkers for clinical conditions. It is often very difficult to determine causal relationships between a given symptom and pathological alterations or contributing factors in clinical studies, but animal models enable the examination of direct causal relationships between behavioral and neurobiological abnormalities. The detailed neuropathology and the etiology of BPD are very complex and are only partially understood; hence, valid animal models for BPD are urgently needed and might offer crucial insights into the understanding of basic neurobiological processes. However, the development of suitable animal models of psychiatric disorders poses major challenges [177, 178]. Generating a valid and potentially holistic animal model requires profound knowledge of the etiology, pathogenesis, and pathophysiology of a given disorder, and this detailed knowledge is not available for many neuropsychiatric conditions. Multiple and varied causal factors may induce similar phenotypes [177], raising further complications.

The difficulties in establishing valid animal models in neuropsychiatric research become particularly apparent in the study of personality disorders, which are associated with ways of thinking and feeling about the self and others that significantly and adversely affect how an individual functions in many aspects of life. Many of these features are uniquely human and can only be inferred with strong limitations in rodent models. Of the core behavioral traits of BPD, only some sub-aspects, such as emotional reactivity toward aversive/appetitive events, impulsive behavior, pain processing, social competence, and social needs (e.g., social recognition, social trust, and the incentive value of social contact), can be assessed with adequate face validity in rodents (e.g. [179–181]). In particular, an aberrant social endophenotype can be modeled and examined with high validity in laboratory rodents (especially in rats) since rats and mice are highly social, have complex social structures, and express a rich repertoire of behavior patterns used for social recognition, affiliation, sex, and aggression [179, 182].

Recently, a novel rat model was established for social rejection in order to assess the acute and long-term consequences of such adverse peer-experiences in adolescence at both the behavioral and the molecular level [183]. Since BPD patients often show an augmented sensitivity toward social rejection [184], this model is also of relevance for BPD research. The model is based on specific social requirements of adolescent rats, which spend more time interacting with peers than do younger or older animals. These peer-directed activities (mostly social play) have a considerable incentive value [185] and are crucial for the development of social competence [186]. In order to model social rejection, adolescent rats of the playful Wistar strain were paired with either a same-strain partner or a less playful Fischer344 strain rat, which is an inadequate social partner for a Wistar rat. Pairing with such an inadequate social partner throughout adolescence was found to decrease later adequate playful peer-interactions for Wistar rats without depriving the animals of normal social contact. In the long-term, these manipulations were found to increase the pain threshold and emotional reactivity in these animals and to concomitantly induce alterations in corticosterone release and aberrations in the endocannabinoid system in the amygdala and the thalamus [183].

## Conclusions

Clinical, phenomenological, and experimental findings are evidence that emotion dysregulation and maladaptive interpersonal behavior are core features of BPD. Disturbances of emotion processing typically translate into dysfunctional expectations and interpretations in the context of social interactions, and vice versa. On a neuroregulatory level, this could be manifested through disturbances of the hierarchically modulated prefrontal and prefrontal-amygdalar control circuits. Consequences of these dysregulations include intense aversive tension (which often leads to reduced executive functioning), temporary disruption of integrative psychic functions (dissociation), and the activation of learned maladaptive coping strategies. From an interpersonal perspective, the most robust findings are dysfunctional interpretation of social cues, hyper-mentalization, problems with coaxing, and hypersensitivity to social rejection and threat. Each of these dysfunctional social cognitions is closely linked to the emotional system, resulting in intermittent experiences of intense fear of abandonment, social rejection, and a strong desire for unconditional love.

## Where do we go from here?

As shown above, the model of close interaction between emotion dysregulation and disturbed social cognition in BPD has been corroborated by a multitude of experimental findings during the last decade. However, important questions still remain. First, we should clarify which of these findings can be identified as prototypical BPD-specific alterations and which can be better characterized as secondary “scars” of life-long chronic stress, medication, or substance abuse. Consequently, we should widen our scope beyond the limits of acute psychopathology. Within this context, we should clarify which of the disturbances observed in adult BPD patients can be traced back to adolescence. Subtle neurobiological alterations might be detectable already in childhood and reveal themselves more strongly in adolescence and early adulthood. Second, as most of the previously published studies did not include clinical controls, we need to establish to what extent psychobiological alterations are specific to BPD. Third, the vast majority of studies has been conducted in female BPD patients, although field trials suggest that males may be equally affected. Fourth, current evidence does not allow for a conclusive interpretation of differential disturbances of neural sub-components involved in emotion processing and social interaction. Finally, the promising attempts to establish animal model for BPD pathology should be continued. In the following sections, we delineate some shortcomings in particular domains of research.

### Pathogenesis and life course

While the pathogenesis of BPD is not yet fully understood, most researchers favor a model that postulates an interplay between genetic predisposition and psychosocial stress during childhood and adolescence. Several studies support that sexual, physical, and emotional traumata manifesting themselves through intense social rejection sensitivity play an important role in the development of BPD [187, 188]. It is unclear whether and how these experiences translate into alterations of neural systems on a neurochemical, functional, and morphological level and ultimately result in disturbed emotion processing or to what extent certain neurobiological characteristics are vulnerability factors for this course and predate the problems in emotional processing. This question could be causally addressed with an adequate animal model. Although some small-scale association studies have been conducted, there is a lack of conclusive data on the potential role of genetics in the development of BPD. This is partly due to the complex interplay of different mechanisms. In particular, progress in genetic research is hampered by a lack of well-defined endophenotypes, i.e., traits with a neurobiological substrate that persist after remission from acute psychopathology [189].

Another field that still contains many unanswered questions pertains to the course of BPD. Evidence from longitudinal studies points to a rapid reduction of the number of fulfilled BPD criteria per individual within a few years, with remission rates of up to 99% at 16-year follow-up [6, 190]. However, this might be misleading since longitudinal studies have also demonstrated that levels of psychosocial impairment do not significantly decline, and that “temperamental” symptoms of BPD such as dependency and anger are more persistent over time [190], even after formal remission according to diagnostic criteria has been reached. A cross-sectional comparison of younger (18–25 years) and older (>45 years) BPD patients showed that, although the prevalence of individual symptoms such as impulsivity, suicidal behavior, and affective instability is lower in older patients, the two age groups are strikingly similar in terms of the average number of fulfilled criteria, Axis I co-morbidities, and functional impairment [192].

### Emotion processing and neuroimaging

As described above, several small-scale studies have tested the reactivity of BPD patients to different emotional stimuli such as aversive pictures or faces. Also, recent studies have begun to measure emotion regulation by, for example, combining aversive stimuli with reappraisal or sensory stimulation. However, a comprehensive analysis of an emotion (dys)regulation network in BPD is lacking. Also lacking are studies that compare different types of emotion regulation paradigms within the same individuals, as well as studies that investigate the influence of treatment (e.g., psychotherapy) on measures of emotion regulation.

At the current stage of research, the interpretation of volumetric and spectroscopic results is difficult for several reasons. Study samples have often been quite small and have contained subjects with various co-morbidities. Methodological differences between the studies need to be considered, including a lack of differentiation of functionally different subregions within the amygdala, the ACC, and the OFC, as well as differences with respect to field intensities and measurement reports (including voxel size). Furthermore, many studies have included patients who were being treated with psychotropic medications, some of which are thought to have a substantial impact on structural alterations. None of the studies conducted so far has been able to find links between volumetric findings and psychopathologically relevant disturbances of emotion regulation.

### Social interaction

Social interaction can be described as a dynamic sequence of actions between individuals during which the behavior of one participant is continuously modulated by the past actions and anticipated future actions of another. Early studies suggested that the objective behavioral and neurobiological description of this interaction behavior allows for a new perspective on interpersonal dysfunctioning in BPD. However, the number of studies that use such an approach is still very small, and it is unknown how their data fit with findings from self-based and observer-based questionnaires that provide information on how interaction behaviors are subjectively perceived. Furthermore, a successful social encounter is based on the integration of a multitude of social cognitive processes, and a dysfunction of any of these may lead to interpersonal disturbances. Although much attention has been focused in the past on the investigation of impairments of single social cognitive processes in BPD, it is still unknown to what extent these described alterations contribute to the dramatic problems in the social lives of these patients. Thus, future studies need to manipulate these processes experimentally in the context of interactions in order to gain further insight into the mechanisms of impaired interpersonal relations and to enable the design of specific therapeutic intervention strategies.

### Animal models

Animal models, such as the Wistar/Fischer rat model for social interaction described above, have the potential to shed further light on the basic neurological circuits and processes involved in rejection experiences during adolescence. Rodent models addressing discrete symptomatic aspects of BPD are quite useful; however, to date, no holistic animal model has acounted for the etiopathology, developmental aspects, pathogenesis, and complex symptomatology of the disorder. Although the detailed etiology of BPD is still only partially understood, a history of early aversities appears to be closely connected to BPD (for review, see [188, 193, 194]). Social experiences throughout the life span may interfere with gene expression, brain development, and behavior, and it has been shown that these influences have a particularly profound effect during early development. In mammals, mother-infant interactions are the primary source of tactile stimulation for the developing offspring. These stimulations influence not only physical growth but also various neurodevelopmental processes [195–198], and laboratory rodents and nonhuman primates have shown that variances in maternal care behavior induce lasting neurobiological changes and affect offspring phenotype (for review, see [197, 198]).

## A research agenda for BPD and ED

In our view, the overarching aim of BPD research over the next ten years should be to elucidate central pathomechanisms of emotion processing and social interaction in BPD parallel on the subjective, behavioral, and neurobiological levels. Ultimately, the clarification of these central pathomechanisms should improve strategies for primary and secondary prevention and help to optimize assessment and treatments on both a psychotherapeutic and a pharmacotherapeutic level. The first step should be for researchers to investigate central pathomechanisms (disturbed emotion processing and its implications on social interaction) with respect to BPD specificity, age dependence, gender dependence, and long-term stability beyond remission of acute BPD symptomatology. The second step should be to seek validation of identified key mechanisms as potential endophenotypes and to use these to tailor specific therapeutic interventions.

One approach could be to focus on core psychobiological mechanisms in the complex psychopathology of BPD, and to investigate disturbed emotion processing in various facets and at different levels. Some of these mechanisms could potentially be used to define endophenotypes, which should be closer to the site of the primary causes (whether genetic or environmental) than to the diagnostic category of BPD [199]. According to one view [200], endophenotypes for mental disorders are, among other criteria, primarily state-independent, i.e., they manifest themselves whether or not the illness is active. This view also suggests that endophenotypes are related to the development of the disorder and do not mimic long-term consequences or secondary manifestations of co-occurring Axis I disorders. Including remitted patients and adolescent patients in research projects could address these issues.

A major problem in BPD and emotion regulation research is that most studies have been carried out by relatively small teams, which unavoidably results in mono-methodological approaches. A multidisciplinary cooperation would enable the integration of morphological, functional, endocrinological, and neuropsychological paradigms and measurement methods. We have therefore started a large collaborative research program, recently funded by the German Research Foundation as a center grant, which may serve as an example of how to transfer a research agenda into a multifaceted and translational program. The central aim of this program, titled “Clinical Research Unit (CRU): Mechanisms of Disturbed Emotion Processing in Borderline Personality Disorder”, is to break down the emotion processing and interactive problems of BPD on a psychobiological level and to elucidate the underlying mechanisms. The results will be used to fine-tune and expand currently existing psychosocial treatments and to develop innovative pharmacological approaches. The following presents an overview of the CRU and delineates its major aims and methods.

**Figure 1:**
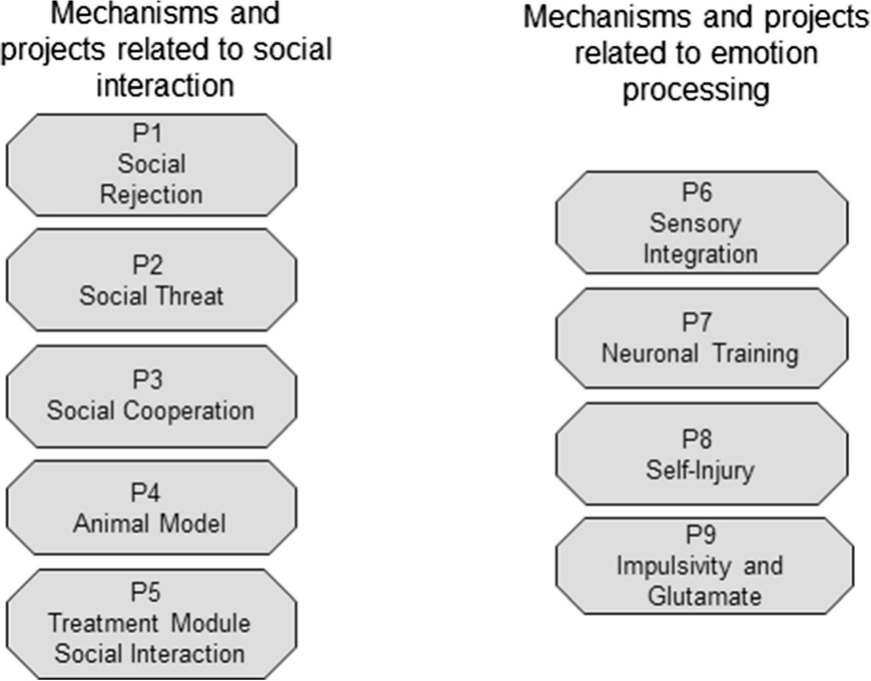
**Overview of the mechanisms and projects of the CRU related to social interaction (left) and emotion processing (right).**

The CRU consists of nine individual projects (see Figure 1). Projects **P1 to P5** focus on psychophysiological and neural parameters related to disturbed social interactions and their implications for emotion regulation, as follows:`

- ***P1*** investigates behavioral and neurobiological patterns in response to experimentally induced social exclusion in acute adult and adolescent BPD patients and in remitted adult patients. In one sub-project, a virtual reality paradigm is used to induce the experience of group rejection gradually. In a second sub-project, an established rejection paradigm (cyberball) is examined under fMRI conditions.
- ***P2*** comprises two experimental fMRI paradigms and an electrophysiological paradigm, comparing acute adult and adolescent BPD populations and a remitted adult BPD population to healthy controls and a clinical comparison group of PTSD patients. It investigates behavioral and neural correlates of BPD patients’ hypersensitivity to social threats and what the implications of the tendency to respond with approach or avoidance. Another issue concerns the aggressive and auto-aggressive manifestations of anger, a prominent feature in BPD. Additionally, this project is the first to systematically consider gender effects in BPD by including male participants.
- ***P3*** stems from the findings of King-Casas and coworkers [167] that shows BPD patients’ substantial deficits in establishing trust with cooperation partners. This topic is being systematically evolved by using the recently developed method of “hyperscanning” with 2 identical audio-visually linked MRI scanners. This methodology allows investigation of dysfunctions in cerebral regions that are related to basic interpersonal interaction and trust and of the ramifications of these dysfunctions on a behavioral level.
- ***P4*** develops an animal model with face validity and construct validity regarding aspects of the etiology and symptomatology of BPD by modulating early social relationships and social interactions in juvenile and adolescent rats. This is intended to induce core symptoms of BPD such as emotion dysregulation, pain sensitivity, and impulse control. The possible lasting consequences of these manipulations on social, emotional, and motivational behaviors are then assessed at different points in life. In order to achieve face-validity for BPD symptomatology, pain perception and stress reactivity are tested. Furthermore, neurobiological alterations that might underlie behavioral changes (e.g., alterations of the HPA axis, the endogenous opioid system, and the endocannabinoid system) are analyzed in order to strengthen the construct validity and possibly in order to lead to pharmacological intervention strategies. A current research aim is to examine behavioral and molecular consequences of differences in maternal care in the context of BPD in laboratory rats. In a later step, these early modulations of mother-infant interactions will be combined with our adolescent social rejection model in order to study potential additive effects of these two adverse social modulations during different developmental periods.
- ***P5*** develops a treatment module designed to specifically improve the regulation of social emotions and interactions of BPD patients, which will be integrated as a further component of Dialectical Behavior Therapy (DBT). It aims to improve adequate interpretations within the social context, mentalization processes, and the building of trust and cooperation.

Projects **P6 to P9** focus on neurobiological parameters of disturbed emotion processing that are primarily located on an intra-psychic (i.e., non-social) level, as follows:

- ***P6*** expands the converging findings on disturbed pain processing and altered body perception in BPD patients, as well as investigating potential disturbances of multisensory interaction. One of its major goals is to discriminate between chronic traits and disturbances resulting from acute stress (dissociation).
- ***P7*** is based of fMRI findings of amygdala hyperreactivity in response to emotional cues in BPD patients. This project expands these findings, and investigates the time course of the blood oxygenation level-dependent (BOLD) response in different brain regions. In a second step, innovative neurofeedback training for BPD using real-time fMRI is established. We assume that it will be beneficial to train patients to control/monitor their neural activity, e.g., by down-regulating limbic hyperactivity.
- ***P8*** investigates an emotion regulation strategy frequently used by BPD patients that involves self-inflicted tissue injury. In one sub-project, the affective components of tissue-damaging and non-tissue-damaging pain are investigated. In a second sub-project, following stress induction, incisions will be applied while psychophysiological, neuronal, and neurochemical parameters of emotion regulation are assessed. It is hypothesized that reduction of stress levels will be stronger following tissue-damaging when compared to non-tissue-damaging pain.
- ***P9*** uses spectroscopy to assess glutamate and GABA metabolism in the ACC and also considers the impact of acute stress on executive functions in BPD.

Overall, more than 1000 potential participants are screened, and approximately 300 patients with current or remitted BPD are included in one or more of the above-mentioned projects. Patients are screened and allocated to the individual projects via a central core project which also conducts diagnostic assessments. Of course, this research program can only cover some areas that are of relevance in the field of BPD. Besides this program, other aspects of research certainly deserve more attention in the field of BPD, e.g. large longitudinal cohort studies or genome studies. However, with this ambitious research program, we hope to answer several of the open research questions described above and to be able to pave the way for tailoring individualized treatments for patients with BPD.

## Acknowledgements

The authors would like to thank the German Research Foundation (DFG) for funding.
